# A Pipeline NanoTRF as a New Tool for *De Novo* Satellite DNA Identification in the Raw Nanopore Sequencing Reads of Plant Genomes

**DOI:** 10.3390/plants11162103

**Published:** 2022-08-12

**Authors:** Ilya Kirov, Elizaveta Kolganova, Maxim Dudnikov, Olga Yu. Yurkevich, Alexandra V. Amosova, Olga V. Muravenko

**Affiliations:** 1 All-Russia Research Institute of Agricultural Biotechnology, Timiryazevskaya Str. 42, Moscow 127550, Russia; 2 Moscow Institute of Physics and Technology, Dolgoprudny 141701, Russia; 3 Engelhardt Institute of Molecular Biology, Russian Academy of Sciences, Moscow 119991, Russia

**Keywords:** satellite DNA, Nanopore sequencing, genome, tandem repeats, pipeline

## Abstract

High-copy tandemly organized repeats (TRs), or satellite DNA, is an important but still enigmatic component of eukaryotic genomes. TRs comprise arrays of multi-copy and highly similar tandem repeats, which makes the elucidation of TRs a very challenging task. Oxford Nanopore sequencing data provide a valuable source of information on TR organization at the single molecule level. However, bioinformatics tools for *de novo* identification of TRs in raw Nanopore data have not been reported so far. We developed NanoTRF, a new python pipeline for TR repeat identification, characterization and consensus monomer sequence assembly. This new pipeline requires only a raw Nanopore read file from low-depth (<1×) genome sequencing. The program generates an informative html report and figures on TR genome abundance, monomer sequence and monomer length. In addition, NanoTRF performs annotation of transposable elements (TEs) sequences within or near satDNA arrays, and the information can be used to elucidate how TR–TE co-evolve in the genome. Moreover, we validated by FISH that the NanoTRF report is useful for the evaluation of TR chromosome organization—clustered or dispersed. Our findings showed that NanoTRF is a robust method for the *de novo* identification of satellite repeats in raw Nanopore data without prior read assembly. The obtained sequences can be used in many downstream analyses including genome assembly assistance and gap estimation, chromosome mapping and cytogenetic marker development.

## 1. Introduction

Satellite DNA (satDNA) consists of multi-copy tandemly organized repeats (TRs) and can comprise a substantial portion of most eukaryotic genomes [1]. Although satDNA was discovered more than 60 years ago, it is still an enigmatic part of eukaryotic genomes, and its current function in the cell is still a matter of debate [2]. It was shown that TRs play multiple important roles in a number of biological processes including cell division, gene expression regulation and genome architecture [1,3,4,5]. The well-known examples of functionally important TRs are centromeric repeats, which have been identified and elucidated in a number of species [4,6]. Centromere repeats are bound with the centromere histone H3 (CENH3) and involved in centromere location establishment as well as proper chromosome segregation during cell division [6]. TRs have been an essential part of all plant genome studies to date, and they have been shown to be involved in plant genome evolution and speciation [7,8,9].

Since TRs form long arrays in the genome, they can be relatively easy visualized by fluorescent in situ hybridization (FISH) [10,11,12,13,14,15,16]. This property of TRs has been broadly exploited in plant cytogenetic studies to develop chromosome markers, which are useful tools to trace individual chromosomes during cell division, and also to study chromosome evolution and rearrangements [9,11,12,13,17,18,19,20,21,22]. Detection of some TRs by FISH can be achieved even without a denaturation step and long hybridization, allowing for a quick (few hours) chromosome identification [13,19,20,21]. TR-based FISH karyotyping has been established for many plant species including model organisms and crops [11,12,13,16,19,20,21,22]. FISH-mapped centromeric TRs are also a valuable resource for the integration of chromosome maps and genome assembly, as well as for the validation of chromosome-level genome assembly [10].

The discovery of new TRs has for a long time been based on ‘wet’ lab methods, including density centrifugation [23] and genomic DNA restriction [24,25,26]. Although these methods played an important role in the initial elucidation of TR composition and chromosome localization, they are technically challenging and do not provide information on all the TRs in a genome. An alternative group of methods exploits genome assembly data for the TR search; these methods are based on a representative of string-matching algorithms, e.g., Tandem Repeat Finder [27], nucleotide autocorrelation functions [15] or Fourier transformation [28,29]. The results of all these computational methods strongly depend on the quality and contiguity of the genome assembly. However, repetitive sequences are often significantly underrepresented or collapsed in the genome assembly, and therefore, assembly-based methods for TR identification usually underestimate the TR copy number in the genome [10,30,31]. The real breakthrough in satellite DNA studies was the development of methods based on the analysis of next-generation sequencing data [32,33]. These methods performed similarity-based read clustering, *de novo* repeat family identification and annotation, as well as repeat sequence assembly. Moreover, deep characterization of repeatome composition and evolution have been carried out in a number of species [9,10,13,34,35,36,37,38]. However, TR identification based on short-read NGS data lacks information about the organization of repeats at the genomic scale. At the same time, the genome context of TR location is important for understanding the origin and evolution of new TR families [14].

The introduction of Oxford Nanopore Technology (ONP, Oxford, UK) sequencing has revolutionized the field of genomics, enabling high-throughput long-read sequencing at a low price with the use of portable sequencing machines (MinIONs). In the context of tandem repeat research, raw ONP reads provide a backbone for sequencing long arrays of tandem repeats and studying the genomic context of TR organization [14,39,40]. In addition, ONP data can also be used to decipher the epigenetic profile of tandem repeats. Currently, several algorithms have been proposed to identify TRs in individual ONP reads, such as TideHunter [41] and NCRF [42]. However, *de novo* identification of novel high-copy TRs from raw ONP data obtained by low-depth genome sequencing and their classification into families, as well as the estimation of total genome abundancy, are still not straightforward. Moreover, this type of the data is rapidly accumulated in databases. In this study, we present NanoTRF (https://github.com/Kirovez/NanoTRF, accessed on 3 June 2022), a computational pipeline for the *de novo* identification, quantification and consensus assembly of high-copy TRs in raw and low-depth Nanopore sequencing data.

## 2. Results

### 2.1. Description of NanoTRF

**Figure 1:**
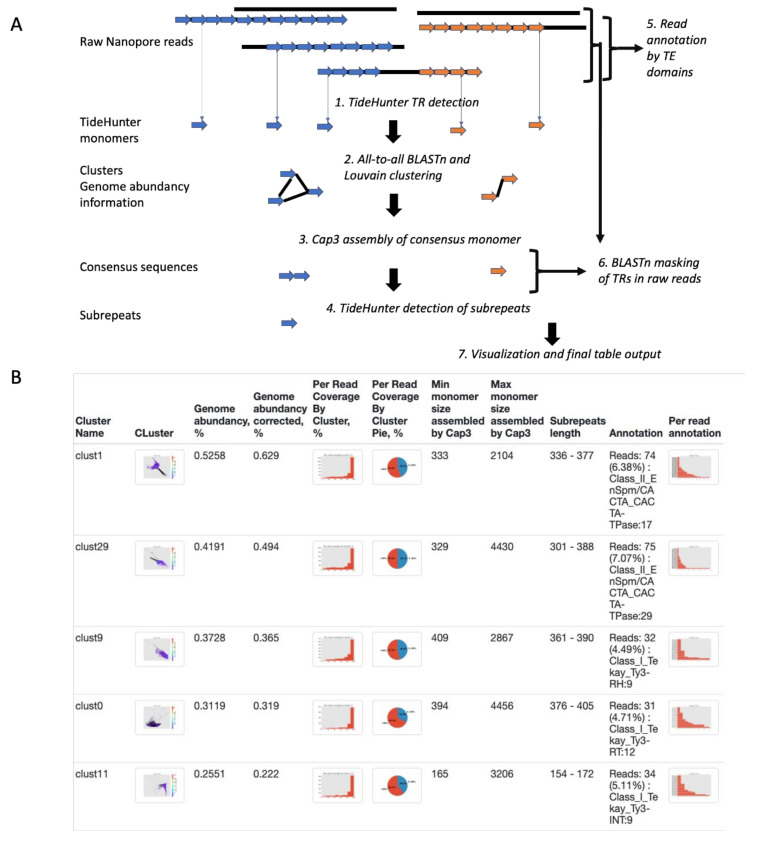
Schematic representation of the NanoTRF pipeline and the report. (**A**) The scheme showing the seven main steps in the NanoTRF pipeline: (1) TR identification in individual ONP reads by TideHunter; (2) all-to-all similarity search between TRs and clustering of highly similar TRs; (3) consensus monomer assembly by cap3; (4) detection of subrepeats in the contigs; (5) ONP read annotation by the TE protein database; (6) calculation of ONP read coverage by TRs and (7) final report generation. Monomers of distinct TRs are colored in orange and blue. (**B**) A screenshot of the output table from the html file generated by NanoTRF.

The key aim of this pipeline is the identification of high-copy tandem repeats (TRs) and the reconstruction of their consensus sequences. The only input required by NanoTRF is the raw Nanopore read file in fastq or fasta format from low-depth (it was tested on 0.1–1×) genome coverage sequencing. NanoTRF includes several steps (Figure 1): (1) TR detection in individual raw Nanopore reads by TideHunter software [41]; (2) an all-to-all similarity search between identified single-read TR sequences using BLASTn [43] and clustering of single-read TRs followed by community detection using Louvain heuristics and TR genome abundancy calculations; (3) TR consensus monomer assembly by cap3 [44] for each cluster; (4) TideHunter analysis of individual consensus and detection of subrepeats; (5) a similarity search of nanopore reads carrying TRs from each cluster with TE protein domains; (6) conversion of BLASTn consensus monomer sequences to raw reads for calculation of the percentage of reads similar to the TRs; (7) Draw cluster layout and read annotation (pie chart and histogram of read coverage by TRs and read annotation by TE domains) figures and writing the final report table. NanoTRF reports several files: (i) a fasta file possessing consensus contig sequences of TRs assembled from monomers in individual clusters by cap3; (ii) a summary table containing per-cluster information and (iii) a html report with general information about TRs, graph layouts, figures, read coverage by TRs and read annotation by TE domains.

### 2.2. TR Abundancy Calculated from Long (NanoTRF) and Short (TAREAN) Reads Are Well Correlated

To validate the NanoTRF pipeline, we performed Nanopore sequencing of the genome of *Deschampsia antarctica* E. Desv. (Poaceae), a species with a well-characterized satellitome composition [9,38]. In total, we obtained 1,452,313 reads with N50 1305 bp and a total number of bases of ~4 Gb, representing about 0.8× of coverage of the *D. antarctica* genome. In total, 43 highly abundant TR clusters (genome abundancy 0.52–0.01%) with a median monomer length of 390 bp (42–2192 bp) were identified by NanoTRF (Supplementary File S1). Based on the genome abundancy of each TR, we estimated that 3.5% of the *D. antarctica* genome is occupied by satellite DNA.

**Figure 2:**
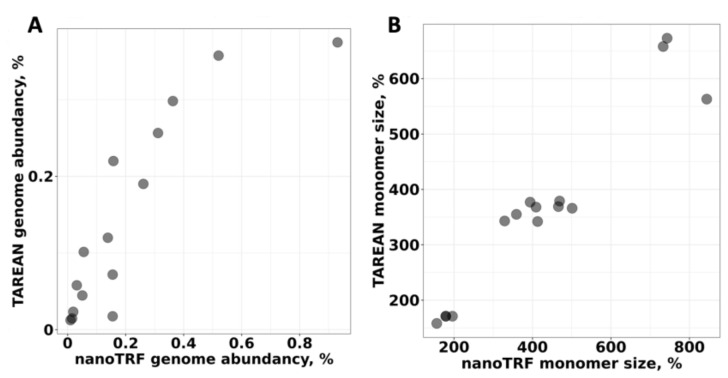
Comparison of the results of the NanoTRF and TAREAN identification of TRs in the *D. antarctica* genome based on the (**A**) genome abundancy and (**B**) monomer length. Individual TRs are represented as black dots. X-axis and Y-axis show the results from NanoTRF and TAREAN, respectively.

We also compared the NanoTRF results with the results from the TAREAN software [33], which uses Illumina reads. For this, we performed identification of TRs by TAREAN using publicly available Illumina NGS reads for *D. antarctica* [37]. The comparison of TRs found by NanoTRF and TAREAN revealed that 93% (15 of 16 high-confident TRs) of the TRs identified by TAREAN were also detected by NanoTRF. Based on the genome abundancy of each TR found by TAREAN, we calculated that 2.44% of the *D. antarctica* genome is occupied by satellite DNA. This value is in good accordance with the NanoTRF results (3.5%). We further compared the genome abundancy, and the monomer length calculated for TRs found by NanoTRF and TAREAN resulted in a good correlation (correlation coefficients were 0.87 and 0.95, respectively (*p*-value < 1.1 × 10^−5^; Figure 2A,B). Similarly, alignment of the monomer sequences assembled by NanoTRF and TAREAN revealed that most of the sequences had >93% similarity.

Thus, our results demonstrated that NanoTRF is a robust tool for the *de novo* identification, sequence assembly and genome abundancy prediction of high-copy tandem repeats from raw ONP data.

### 2.3. NanoTRF Data for Clustered and Dispersed TRs

On the genomic level, TRs can be dispersed over chromosomes or can generate long arrays. TRs that are organized in megabase-sized arrays are usually a part of centromeres, subtelomeres or heterochromatin regions of plant chromosomes. The ability to distinguish between dispersed and clustered TRs is an important task.

**Figure 3:**
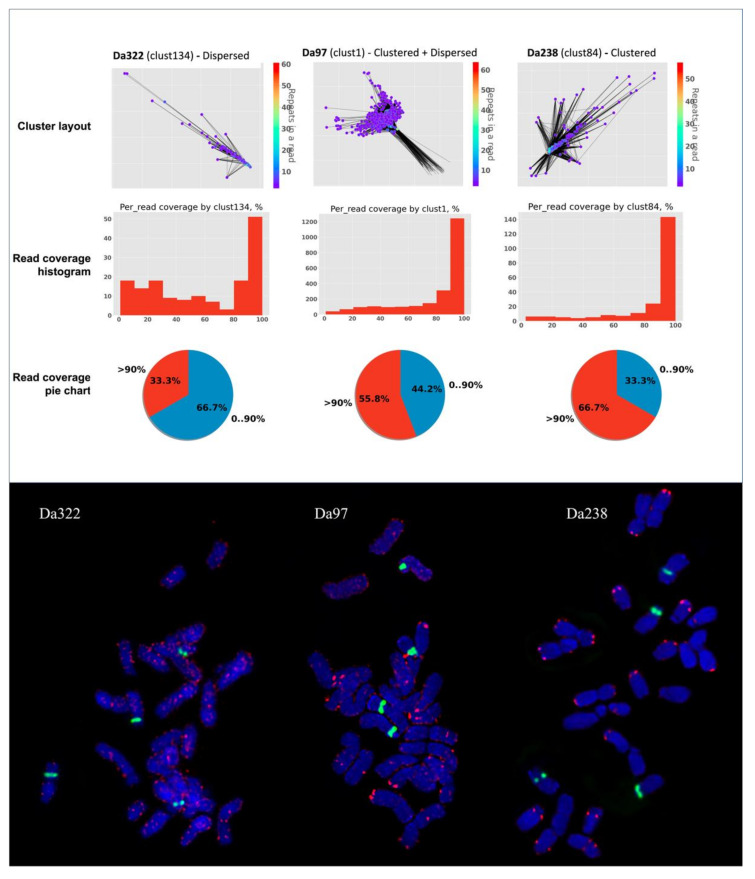
Comparison of the cluster layouts and read coverage data of three TRs with different FISH patterns on chromosomes of *Deschampsia antarctica*: Da322 (dispersed), Da97 (dispersed and clustered) and Da238 (clustered) probes. Bottom picture shows the results of FISH experiments with labeled individual TRs (Da322, Da97 and Da238; red fluorescence signals) and 45S rDNA (green fluorescence signals). Chromosomes are stained by DAPI (blue fluorescence signals).

To achieve this, NanoTRF provides data on the percentage of the sequence of individual Nanopore reads that shows similarity to NanoTRF clusters. This data is presented as a histogram and a pie chart in the NanoTRF html report, and it can be useful for the estimation of the relative TR array size. To validate this, we compared the read coverage using three TRs of *D. antarctic—*Da322, Da97 and Da238—having dispersed, dispersed and clustered and clustered chromosome organization, respectively (Figure 3). We found that the majority of reads in the clusters of all three repeats were covered by a TR of >90% (Figure 3). However, the percentage of the reads covered by less than 90% of their sequence (Figure 2) by the corresponding TR was 2 and 1.5-times higher for the dispersed (Da322) and dispersed + clustered (Da97) TRs, respectively, compared to the TR with a clustered chromosome organization (Da238).

These results showed that the read coverage data provided by NanoTRF can be used to estimate the chromosome organization of TRs and also to select TRs that are suitable for molecular cytogenetic studies as chromosome FISH markers.

### 2.4. Cluster Annotation Showed an Association between TR and Transposable Elements

Previous reports on some plant species, e.g., *Lathyrus sativus* L. (Fabaceae), have suggested that TR origin and evolution can be tightly connected with the amplification of certain TE families, including Ogre elements [14]. NanoTRF performs automatic annotation of reads in each cluster with the use of the TE protein domain database. This information can be useful for dissecting TR–TE coevolution events.

**Figure 4:**
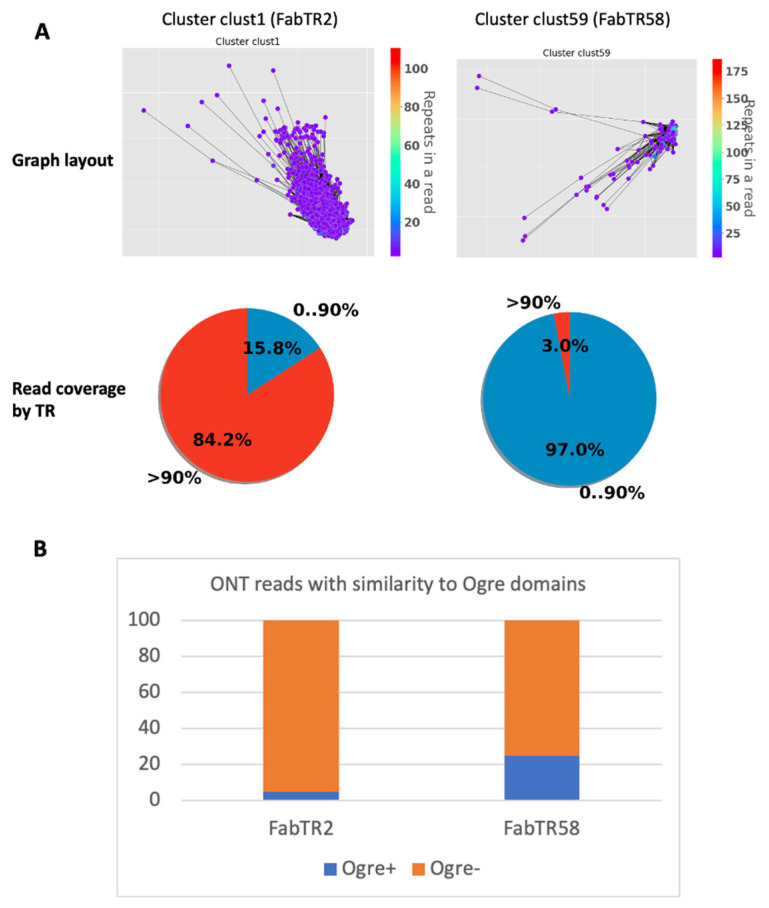
NanoTRF results for the analysis of the FabTR2 and FabTR58 repeats. (**A**) Graph layouts and pie chart showing the percentage of reads with different TR occupancies for clusters clust1 (FabTR2) and clust59 (FabTR58). (**B**) Bar plot of the percentage of reads in the two clusters (clust1 and clust59) possessing similarity to Ogre TE domains.

To assess this, we performed a NanoTRF analysis of a subset (total read length—500 Mb) of ultra-long reads of *L. sativus* that were previously used for tracing TR origins using Ogre elements. Using the similarity search, we identified the NanoTRF clusters corresponding to Fab TRs of *L. sativus*. Among them, FabTR2 was shown to generate long-arrays in the genome that were occasionally disturbed by Ogre insertions [14]. Similarly, the NanoTRF cluster of FabTR2 (clust1) had only 15.8% of its reads partially (<90%) covered by this TR—a feature corresponding to clustered TRs (see above). Additionally, 5% of the reads in this cluster showed similarity to domains of TEs, including Ogre elements, corroborating with occasional insertions of these elements into FabTR2 arrays. Opposite to FabTR2, another TR—FabTR58—was shown to be mostly part of Ogre element copies [14]. Indeed, the analysis of the corresponding NanoTRF cluster (clust59) also showed that this repeat does not generate long arrays in the genome, as follows from the very low number of reads (3%) with >90% coverage by the TR (Figure 4A). Besides this, 25% of the reads possessed a similarity to Ogre element protein domains, which is five-times higher than for the FabTR2 cluster (Figure 4B)—supporting the previous conclusion of the frequent co-occurrence of FabTR58 and Ogre elements in the genome.

These results demonstrate that read annotation by TE domains and read coverage by TRs provided by NanoTRF are useful for the elucidation of TR–TE co-location in the genome.

## 3. Discussion

Satellite DNA is an important component of many eukaryotic genomes including human and plants. In plant genomes, satellite DNA is presented in a large number of families and with wide diversity, and it plays an essential role in organizing the structural integrity and functioning of genomes [1,4,5]. TRs exhibit remarkable diversity in monomer size, genome abundancy, chromosome localization and sequence composition, even between closely related species [3,13,16,45,46]. During the last decade, the number of discovered satellite tandem repeats (TRs) has rapidly accumulated because of new tools developed for *de novo* TR identification using short reads from NGS sequencing [32,33]. However, progress in the understanding of the genomic organization of TRs is lagging behind because of challenges in TR assembly and their frequent underestimation in sequenced genomes [30]. At the same time, the long reads obtained by the Oxford Nanopore (ONP) method are useful for the sequencing of long arrays of repetitive DNA [40]. The number of available datasets with ONP long reads is rapidly growing as they can be generated in a conventional laboratory using a portable (e.g., MinION) device and easy library preparation protocols. Theoretically, the length of the ONP reads is only limited by the size of the isolated DNA fragments, making ONP reads an attractive tool for the study of the genomic organization of TRs [14,39,40]. However, the identification of satellite tandem repeats in raw ONP data obtained by low-depth genome sequencing has been hampered by the absence of the user-friendly tools. To fill in this gap, we developed NanoTRF, a simple, robust and easy-to-use pipeline for the direct identification, quantification and analysis of TRs using raw, low-depth ONP data. 

The advantages of NanoTRF are the following: (1) it requires neither prior knowledge about TR sequences nor genome assembly, and therefore it can be used for a broad range of species; (2) even low coverage ONP genomic data (>0.1×) is suitable for NanoTRF; (3) NanoTRF calculates the genome abundance for each TR and the results are comparable with TAREAN [33]; (4) NanoTRF provides additional information on read occupancy by TRs and, as we have shown in the present study, this data can be used for TR filtering according to their genome organization—clustered or dispersed; (5) the read coverage data, together with the read annotation by TE domains, is useful for the elucidation of TR–TE coevolution and genome organization and (6) the html report provides user-friendly access to the NanoTRF results.

Recent studies have utilized ONP reads to elucidate TR genome organization features in plant genomes [10,14,47,48]. The very long size of the ONP reads open the door for the investigation of TRs in individual genomic arrays. Although ONP reads are error-prone, it still possible to detect individual TR arrays in raw reads without contig assembly. This is crucial for satellitome studies as the assembly step may introduce artefacts and significantly shorten the TR array [10]. Previous studies [14] have shown that ONP reads can also provide insights into TR organization on the chromosome level. There can be differences in the chromosome-scale organization of TRs, with some TRs forming long arrays (clustered TRs) while others form short clusters dispersed throughout the genome (dispersed TRs). Not all tandemly organized sequences generate long arrays in the genome [13]. At the same time, clustered TRs are attractive items for molecular cytogenetics and also FISH-based karyotyping. NanoTRF provides information on the portion of ONP reads with similarity to distinct TRs in order to distinguish clustered and dispersed TRs. Using FISH, we demonstrated that NanoTRF reports are indeed useful for separating TRs based on genomic organization. In the case of clustered TRs, this information is useful when selecting TRs for FISH-based chromosome marker design. Another valuable feature in NanoTRF is the TE annotation step in the TR-carrying ONP reads. Modern similarity search algorithms (e.g., DIAMOND [49]) and TE protein database [50] make it possible to detect TE sequences in raw ONP reads with up to 15–20% errors. A previous study demonstrated that this approach can be used to decode TE–TR coevolution events [14]. By using NanoTRF, we have demonstrated that TE–TR coevolution events can now be detected automatically. 

In conclusion, NanoTRF introduces a new modern toolbox for TR analysis using Nanopore raw genomic sequencing data in plants and other eukaryotes.

## 4. Materials and Methods

### 4.1. Overview of NanoTRF

NanoTRF is written in Python 3 and depends on several libraries including matplotlib (https://matplotlib.org/, accessed on 3 June 2022), biopython [51], networkx [52] and python-louvain (https://github.com/taynaud/python-louvain, accessed on 3 July 2022). NanoTRF includes several steps (Figure 1) and modules. The first module begins with the identification of single-read TRs (‘srTRs’) in individual ONT reads using TideHunter [49] with default parameters. After this, all obtained srTR sequences are subjected to all-to-all similarity comparison by BLASTn [50] with the following optional parameters: -word_size (default = 24), -max_hsps = 1 and –evalue (default = 2). Third-clustering module works using the Louvain clustering method, which groups similar srTRs into communities and attempts to merge similar nodes from similar communities, further building a new network with node communities (srTR clusters). In the next stage, clusters consisting of less than 6 srTRs are removed from further analysis. Next, the module launches the Cap3 software with the additional parameters (-h 100 -n -2 -m 3 -p 80 -s 600) to perform assembly of consensus TR repeats from the srTRs of a cluster. The genome abundancy for each cluster is calculated as follows: $(\sum_{i=0}^{i=n}nrep*len*$ 100)/TRL, where n—number of monomer sequences in a cluster, nrep—number of repeats of the monomer occurs in the read, len—monomer length and TRL—the total length of all input reads. The individual clusters are drawn by networkx [48] and matplotlib libraries. Then, the corrected genome abundancy is estimated by blastn (-word_size 28) masking of raw ONT reads using assembled TRs. The corrected genome abundancy is calculated as the sum of the masked base pairs of the ONT reads by individual TRs divided by the total number of raw ONT reads. The obtained information is also used to calculate the percentage of the ONT reads covered by TRs. Then, the raw reads for which the TRs were clustered are subjected to annotation by DIAMOND [51] and the RExDB [52] database of transposon protein domain sequences. 

### 4.2. Plant Material and DNA Isolation

The seeds of *D. antarctica* (KEW-0521613, St. Georgia, Falkland Is.) were obtained from the Seed Conservation Department of Kew Royal Botanic Gardens (Kew, UK). Genomic DNA was isolated from green leaves using a Genomic DNA Purification kit (Thermo Fisher Scientific, Waltham, MA, USA). The concentration of DNA was assessed with a Quibit 4 fluorometer (Invitrogen, Thermo Fisher Scientific, Waltham, MA, USA).

### 4.3. Library Preparation and Nanopore Sequencing

The library for the ONP sequencing was prepared using an SQK-LSK109 Ligation Sequencing Kit (Oxford Nanopore Technology, Oxford, UK) for 1D genomic DNA sequencing. A MinION (ONT) instrument with an R9.4.1 flow-cell (ONT) was used for sequencing. Sequencing was controlled by MinKNOW v18.07.2 (Oxford Nanopore) and stopped after 48 h. Basecalling was carried out by Guppy software v 4.0.11 (Oxford Nanopore Technology, UK).

### 4.4. Search of TRs in Illumina Data by TAREAN Software

Publicly available Illumina reads of *D. antarctica* [37] were downloaded from NCBI (accession number—SRR1158316). Quality checks and adapter trimming were performed by the FastQC (https://www.bioinformatics.babraham.ac.uk/projects/fastqc/, accessed on 1 May 2022) and Trimmomatic [53] tools, respectively. TAREAN [33] software was run as a part of the RepeatExplorer pipeline [32].

### 4.5. Fluorescence in Situ Hybridization

In the FISH assays, we used two wheat DNA probes: pTa71 enclosing 18S-5.8S-26S (45S) rDNA and pTa794 containing 5S rDNA [54]. These DNA probes were labelled directly with fluorochromes Aqua 431 dUTP or Red 580 dUTP (ENZO Life Sciences, NY, USA) by nick translation according to the manufacturers’ protocols. Additionally, oligonucleotide probes Da322, Da97 and Da238 were designed based on the satDNA sequences of Da322, Da97 and Da238 (Table 1). These probes were produced and labelled directly with Cy3-dUTP in *Syntol* (Moscow, Russia). FISH procedures were performed as described previously [9].

## 5. Conclusions

Long reads provide indispensable information on repetitive DNA organization. However, the exploiting of raw ONP data obtained by low-depth genome sequencing for high-copy tandem repeat discovery has been hampered by the absence of user-friendly tools. In the present study, we developed NanoTRF, a pipeline for TR repeat identification, characterization and consensus monomer sequence assembly. NanoTRF only requires a raw Nanopore read file from low-depth (<1×) genome sequencing. An informative html report and figures on TR genome abundance, monomer sequence and monomer length, as well as annotation of transposable elements (TEs) sequences within or near TR arrays is generated. The obtained TR sequences can be used in many downstream analyses including genome assembly assistance and gap estimation, chromosome mapping and cytogenetic marker development. We believe that NanoTRF will significantly accelerate the progress in satellitome research as it opens the way for rapid TR identification, placing individual TRs into their genomic context.

## Figures and Tables

**Table 1:** Sequences of the TRs of *Deschampsia antarctica* used for generation of the oligonucleotide FISH probes.

Tandem Repeat/Genome Proportion, %	Length, bp	Sequence
Da 97/0.21	342	CCCACGGGCTAGGGTTTCGCTGGAAAAGTACCGCCGGAGCGCCGGAATCCCACGAAAACTTGCGTGTGGCCCTAGCATGCATGCACAAGTGTGGTGGAAGGTTCCTAGATGCAATACGTAGCTCCCGGGTGCGATCCTGTTCGCGCGCATGCGATAACACTTAGAAAACTGCTGGACCTCTGGGAGGAATCTCCCGCTACGGGTCAAACGGAGGGCAACCGGTGGAATCATGGGCCCAACCTTGGTTTTCCATGTAGATATGCCCTAAACAAACCCAAACCAACAAAAAAGTACATTGGTCAACCCTCGTACGGAGAATGCTAGGGGGCTAGACCTGCGGGT
Da 238/0.042	379	GCCTAACACCCTATCGTAGACACCCATGGGTTGGGGCGCAGTGCACGTAATACTATACGGATCCAGCGTTCCATCGAATTTTGAGTTTTTACTGCAGAAACTTCCATTTTTCCTAGACTTGTGAGCACTTTTTGAGGCCCTAAAAAGGCTTTTTTGGGGTCGAGATGGTCCGCACGCGTGCTGGGGTGTGTGCACGTATGTAAAATCATCCGGATTGCAAAAATTAGAAGTCCTTTTATC CTAGTTCTCCGAGATCTTTCTAACGCCTTCGAAACCGCCTCAATCGGAGCTCGTTCTCATTCGCGTCGTTAGTATTAACAAAGTTCCTCCGTACGATGATCCTTTGCTTTCAACGGTCACCGTTTCTTCTCAGGCGTGA
Da 322/0.013	342	GGTCTAGGGTTTCCCCGGATACAGACCACCGGAGCGTCGGAATCGCTGGAAAACTTGCATGTGTCCCTAACATATGTGTACAAGTGTGATGTAAGGTTGGTAGATGGCATATCTAGGTCCCAGGCGTGACGCTGTTCGCAGACATGGGCTAACACTTGGTAAAATCCTGGATCTGTATGTGGAAACTCCCGCTACGGGTCAACCGGAGCCTATTTTATGGTAAAGTAGGCCCAACCTCTGCTTTCCATGTACATATGTCCTAAACAAACCAAAACAAGGAAAAAACTCCATTGGTAAACCCTCGTACGGAGAAAGCTATAGGGGTAGATCTGCGGGGTCCCC

## Data Availability

The Nanopore data produced for this study are available in Sequence Read Archive (SRA) NCBI under Bioproject Accession PRJNA708177.

## Supplementary Materials

The following supporting information can be downloaded at: https://www.mdpi.com/article/10.3390/plants11162103/s1, File S1: *D. antarctica* satellitome composition unraveled by NanoTRF.
